# Strain engineering of transverse electric and transverse magnetic mode of material gain in GeSn/SiGeSn quantum wells

**DOI:** 10.1038/s41598-019-40146-z

**Published:** 2019-03-01

**Authors:** Herbert S. Mączko, Robert Kudrawiec, Marta Gladysiewicz

**Affiliations:** Department of Experimental Physics, Faculty of Fundamental Problems of Technology, Wroclaw University of Science and Technology, Wybrzeże Wyspiańskiego 27, 50-370 Wroclaw, Poland

## Abstract

8-band *k* · *p* Hamiltonian together with envelope function approximation and planewave expansion method are applied to calculate the electronic band structure and material gain for Ge_1−w_Sn_w_/Si_y_Ge_1−x−y_Sn_x_/Ge_1−w_Sn_w_ quantum wells (QWs) grown on virtual Ge_1-z_Sn_z_ substrates integrated with Si platform. It is clearly shown how both the emission wavelength in this material system can be controlled by the content of virtual substrate and the polarization of emitted light can be controlled via the built-in strain. In order to systematically demonstrate these possibilities, the transverse electric (TE) and transverse magnetic (TM) modes of material gain, and hence the polarization degree, are calculated for Ge_1−w_Sn_w_/Si_y_Ge_1−x−y_Sn_x_/Ge_1−w_Sn_w_ (QWs) with the strain varying from tensile (ε = +1.5%) to compressive (ε = −0.9%). It has been predicted that the polarization can be changed from 100% TE to 80% TM. In addition, it has been shown that Si_y_Ge_1−x−y_Sn_x_ barriers, lattice matched to the virtual Ge_1-z_Sn_z_ substrate (condition: y = 3.66(x-z)), may ensure a respectable quantum confinement for electrons and holes in this system. With such material features Ge_1−w_Sn_w_/Si_y_Ge_1−x−y_Sn_x_/Ge_1−w_Sn_w_ QW structure unified with Ge_1-z_Sn_z_/Si platform may be considered as a very prospective one for light polarization engineering.

## Introduction

The indirect character of the band-gap in Si and Ge strongly limits the application of group IV semiconductors in light emitters including lasers. However bandgap engineering via strains and/or alloying with other group IV materials (Sn or C) can mark these materials convenient for optoelectronic applications^1–10^. For germanium, the energy difference between the direct and indirect band-gap is quite small (0.14 eV in contrast to Si where this difference equals 2.54 eV) and hence Ge is more favorable than Si for tuning the character of the bandgap from indirect to direct by strain engineering and/or alloying with other elements of group IV.

Using strain engineering and *n*-type doping in the active region, optically pumping^4^ and electrically pumping^8^ Ge lasers have already been reported (*n*-type doping fills up the electronic states in the L-valley up to the Γ-valley and in this way is very favourable for lasing^3^). However, the reliability of these lasers is still not satisfactory. Moreover, it is hard to tune the emission wavelength from such lasers and therefore other approaches are discovered. Alloying Ge with semimetallic α-Sn is one of them^1,2^.

According to theoretical predictions, the incorporation of Sn atoms in Ge crystals cuts the conduction band energy at the Γ-point quicker than at the L-point, leading to a direct bandgap semiconductor at Sn > 5–11% for unstrained GeSn^11–16^. The pioneering experimental work on GeSn alloy have been done by He and Atwater^2^. Up until now a lot of research has been fixated on the growth of high quality GeSn alloys and devices having GeSn alloys^17–29^ and currently the incorporation of Sn atoms into Ge host seems to be the most hopeful approach to reach a group IV direct gap semiconductor as the active region for light emitters^30,31^, which can be integrated with Si platform. The first demonstration of optically pumped direct bandgap GeSn lasers has been described by Wirths *et al*.^32^. In this work the authors have found lasing from bulk GeSn and determined the material gain for this alloy to be ~100 cm^−1^ at the excitation of ~550 kW/cm^2^. Very recently, a demonstration of optically pumped GeSn-based lasers grown on Si substrates and emitting at 2476 nm has also been reported by Al-Kabi *et al*.^33^. These results suggest that electrically pumped GeSn lasers are very close to being available but challenges concerning the operating temperature (T_0_ parameter), emission wavelength, and light polarization still exist for this material system.

In our recent work we have presented that the compressively stained GeSn/Ge quantum well (QW) is a very gifted gain medium for lasers integrated with Si platform^34^. The material gain for such QWs is stronger than for bulk GeSn and can be tuned in a broad spectral range, which is motivating for gas sensing^34^. Since Ge_1−w_Sn_w_ QWs can be integrated with Si platform via Ge_1-z_Sn_z_ virtual substrates, it is also probable to grow tensile strained GeSn QWs when w < z. In this case the built-in strain enhances the direct character of bandgap and due to this, is very satisfactory for laser applications. Alternatively, the material gain spectrum for tensile strained QWs can be dominated by the transverse magnetic (TM) mode since the ground state transition in such QWs is between the electron and the light-hole subband in place of the heavy-hole subband. It suggests that the light polarization engineering via stain should be achievable in GeSn QWs, which is very important for real laser applications and their functionality. However, a careful experimental studies of polarization of light emitted by GaSn QWs have not been carried out up to now. A few reports on gain calculations in GeSn-based QWs can be found in the literature^34–41^ but there are no studies which focus on light polarization engineering in this material system.

In order to show that GeSn QW is a very promising gain medium, which allows to control the light polarization, and to stimulate experimental works in this field, we have calculated the electronic band structure and determined the transverse electric (TE) and transverse magnetic (TM) modes of material gain for the active region composed of Ge_1−w_Sn_w_ QW with the strain tuned from tensile to compressive and Si_y_Ge_1−x−y_Sn_x_ barriers lattice matched to Ge_1-z_Sn_z_ virtual substrate which can be integrated with Si platform. The obtained results are discussed in this paper in the context of recent progress in the growth and characterization of GeSn QWs and possibilities of engineering the electronic band structure and the built-in strain in SiGeSn material system.

## Material Parameters

**Figure 1:**
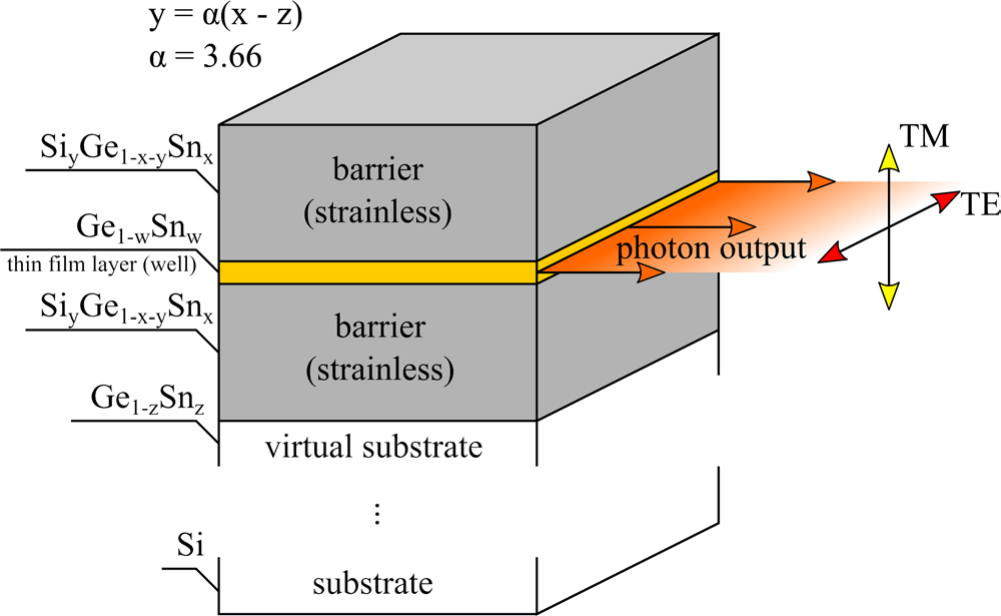
Schematic picture of a Ge_1−w_Sn_w_/Si_y_Ge_1−x−y_Sn_x_ QW deposited on a virtual Ge_1-z_Sn_z_ substrate. The QW is in the region where the in-plane lattice constant ${a}_{\parallel }={a}_{G{e}_{1-z}S{n}_{z}}$.

Figure 1 schematically shows Ge_1−w_Sn_w_ QW with Si_y_Ge_1−x−y_Sn_x_ barriers grown on Ge_1-z_Sn_z_ virtual substrate which is integrated with Si platform. Since the lattice constant in such heterostructures is determined by the virtual substrate, the built-in strain in Ge_1−w_Sn_w_ QW can be tuned from tensile (w < z) to compressive (w > z). In order to ensure the quantum confinement for electrons and holes in Ge_1−w_Sn_w_ QW, barriers with larger bandgap and type I bandgap alignment are needed. In this case Si_y_Ge_1−x−y_Sn_x_ barriers are a very natural candidate, since the incorporation of Si into GeSn opens up the bandgap and allows full strain engineering in this material system. Such barriers can be lattice matched to the virtual substrate and thereby Ge_1−w_Sn_w_ QW can be surrounded by barriers which create the waveguide for the proper wavelength.

The proposed QW structures can be useful in lasers if two conditions are fulfilled. The first condition is determined by the nature of the material, it is a positive material gain for the Ge_1−w_Sn_w_/Si_y_Ge_1−x−y_Sn_x_/Ge_1−w_Sn_w_ QW. The second condition is a good quality of (Si)GeSn materials which is determined by the technology applied and is always a big challenge. In this paper, the first condition is examined, since positive results can strongly motivate to work on the growth of lasers with the active Ge_1−w_Sn_w_/Si_y_Ge_1−x−y_Sn_x_/Ge_1−w_Sn_w_ QW region on Si platform.

In order to calculate gain spectra for Ge_1−w_Sn_w_/Si_y_Ge_1−x−y_Sn_x_/Ge_1−w_Sn_w_ QWs, a credible model and material parameters are required. As shown in our previous papers^42,43^, the 8-band ***k*** · ***p*** model together with envelope function approximation and planewave expansion method are advanced enough to be used to calculate electronic band structures for QWs near the center of the Brillouin zone and determine gain spectra from the calculated dispersion of electron and hole subbands. This model can also be applied to calculate material gain spectra for GeSn QWs if the L valley is properly treated^34^. Thus this model has been applied to study Ge_1−w_Sn_w_/Si_y_Ge_1−x−y_Sn_x_/Ge_1−w_Sn_w_ QWs in this work. Details on 8-band ***k*** · ***p*** Hamiltonian, its elements, and formulas used for calculations of TE and TM mode of the material gain are given in the Methods section.

**Figure 2:**
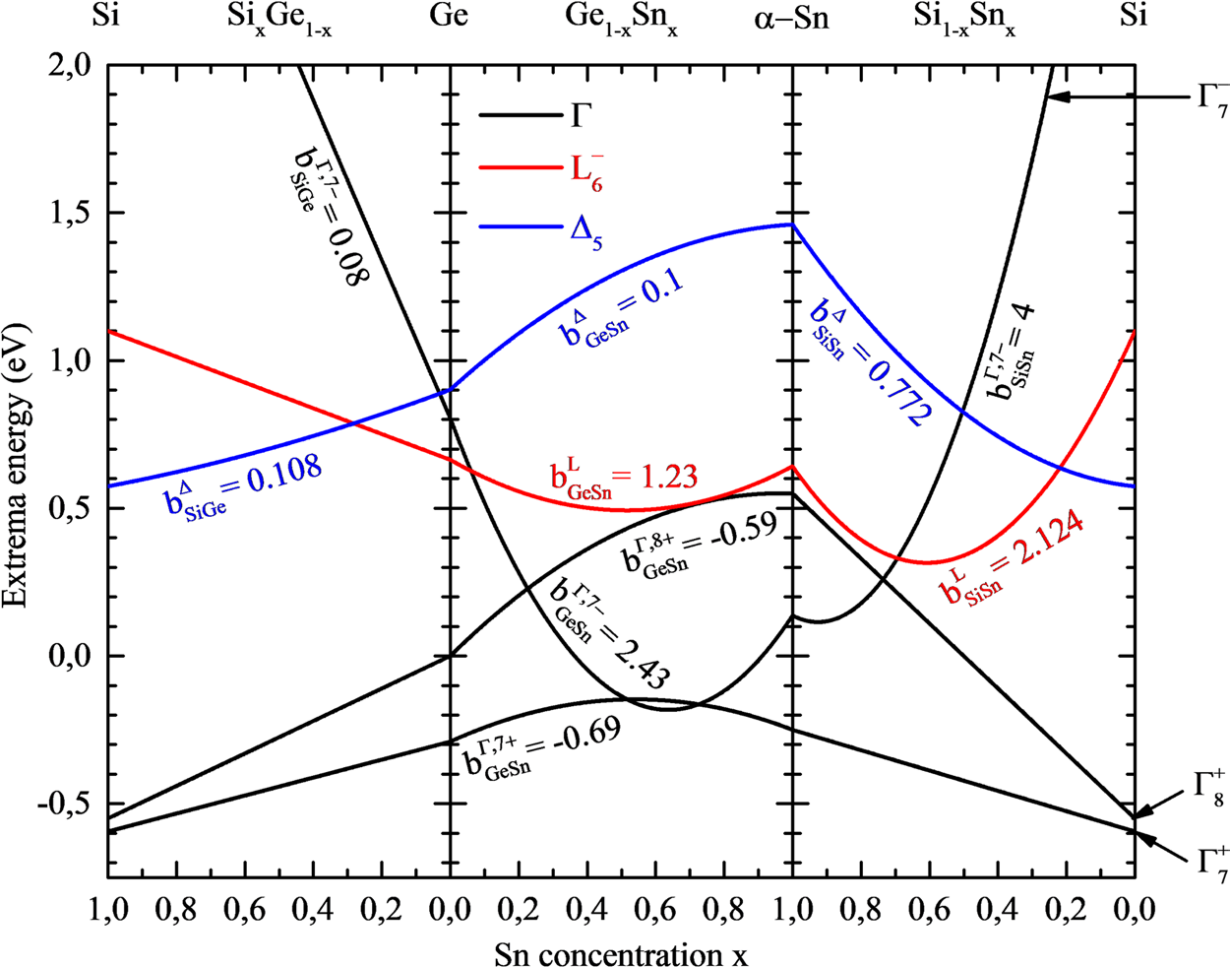
Positions of ${{\rm{\Gamma }}}_{8}^{-}$, ${{\rm{L}}}_{6}^{-}$ and Δ_5_ points in conduction band and positions of ${{\rm{\Gamma }}}_{8}^{+}$ point in the valence band for Si, Ge, and α-Sn together with interpolations and bowing parameters of these positions for the three alloys (SiGe, GeSn, and SiGe).

Figure 2 shows the positions of conduction (${{\rm{\Gamma }}}_{7}^{-}$, ${{\rm{L}}}_{6}^{-}$ and Δ_5_ states) and valence (${{\rm{\Gamma }}}_{8}^{+}$ and ${{\rm{\Gamma }}}_{7}^{+}\,$states) bands in Si, Ge, and α-Sn together with interpolations of these positions for the three alloys (SiGe, GeSn, and SiGe). Nonzero bowing parameters used to calculate the conduction and valence band positions in these alloys are shown in Fig. 2 and the mathematical formula for these calculations is given below

1 $${Q}_{AB}={Q}_{A}(1-x)+{Q}_{B}x-{b}_{Q}x(1-x).$$

In Eq. (1) *Q*_*A*_ and *Q*_*B*_ are the values of the quantity (conduction or valence band position) in the parent material A and B, respectively. The first two terms on right side in Eq. (1) represent the linear interpolation, and the third term is a parabolic deviation from the linearity characterized by the bowing parameter *b*_*Q*_. Bowing parameters for GaSn used in our calculate ons are taken from the recent publication on a comprehensive and detailed study of the composition dependence of lattice constants, bandgaps and band offsets for bulk Ge_1−x_Sn_x_ alloy in the full composition range by using state-of-the-art density functional theory (DFT) methods^16^. These bowing parameters are consistent with the bowing parameters reported in refs^2,11–13,44–49^, where the authors experimentally determined the bandgap bowing for the direct gap to be 2.42 eV^44^, 1.97–2.61 eV^11^, 2.30–2.84 eV^45^, 2.46 eV^46^, and 2.8 eV^2^. In this case it is worth noting that the Burstein-Moss effect plays an important role in GeSn^16^ and this effect can be responsible for some scattering of experimental data. In addition the compressive strain, which is present in GeSn layers grown on Ge substrates (or templates), opens the bandgap and thereby the bowing parameter for the bandgap determined experimentally can be adjusted correctly and lower than the one expected for unstrained GeSn^16^. From first principles calculations the bandgap bowing parameter for the bandgap at the Γ point was reported to be 2.49 eV^48^, 2.55 eV (2.75 eV for Sn 12.5%)^12^, ~2.4–3.0 eV for Sn < 12%^13^, and 3.1 eV^14^. Moreover many authors suggest that the bowing parameter varies with Sn concentration^12,13,47^. It can be another reason for some scattering of bandgap bowing parameters reported in the literature. Taking into account the cited literature data the bowing parameter for the direct gap taken to our calculations (i.e., 3.02 eV^16^) is fully acceptable. The bowing parameter for the indirect gap in GeSn was reported to be 0.89 eV^12^, 0.9 eV^49^, and 1.03^46^, 1.23 eV^11^, 2.28 eV^48^. To our calculations we took the value of 1.23 eV^16^, which is very consistent with the bandgap bowing determined experimentally^11,46^. In this case it is worth noting that the bowing parameters in refs^2,11–14,44–49^ are determined for the bandgap while in ref.^16^ the bowing parameters are determined also for both the conduction and the valence band and thereby they are more useful for our calculations. In addition the band offset between Ge and Sn is determined in ref.^16^ and therefore it is preferred to adopt the band offset and bowing parameters from one reference where they are determined within one consistent approach. In this case it is also worth noting that the band offset reported in ref.^16^ is quite close to the band offset reported in ref.^12^ (0.53 vs. 0.91 eV). For SiGe the bowing parameter for the direct gap has been assumed to be zero in order to be consistent with the recent article on this issue^50^. For SiSn the bowing parameter for the direct gap is assumed to be 4.0 eV according to ref.^51^. In this case it is worth noting that larger bowing parameters are expected for SiSn than for GeSn since Sn differ more significantly from Si than from Ge in terms of atom size and electronegativity. This difference also suggests that the empirical formula with the constant bowing parameter (i.e., Eq.(1)) can be invalid for this alloy in full content range since the bowing parameter can vary with Sn concentration. So far this issue is not explored very carefully but larger bowing parameter for the direct gap in SiSn (13.2 eV) was suggested by D’Costa *et al*.^52^ from the analysis of results for SiGeSn with low Sn concentration. The lower value of bowing parameter selected to our calculations is fully justified since we are considering SiGeSn alloys with larger Sn concentration which are close to those which are reported in ref.^51^. In case of SiSn the whole bandgap bowing is incorporated into the conduction band since its distribution between the conduction and the valence band is unknown at this moment (note that it is a typical first approximation in semiconductor alloys in such a situation).

Remaining material parameters for (Si)GeSn alloys, which are needed for the electronic band structure and the material gain calculations, are obtained from the linear interpolation of material parameters for the parent materials (Si, Ge, and Sn) taken from refs ^16,48,53–69^ and are given in the Table 1. Currently it is experimentally documented that the lattice constant of GeSn is described by the linear interpolation^70^. This conclusion is also very consistent with the recent DFT calculations of the lattice constant for GeSn^16^. For remaining parameters (elastic constants (C_11_ and C_12_), deformation potentials, etc.) it is expected that the deviation from linearity of interpolation will be negligible like in other semiconductor alloys^71^. Even if there is significant nonlinearity for given parameter, but its value is unknown, the best solution is to take the linear interpolation as the first approximation and such solution is applied in our case.

**Table 1:** Material parameters for Si, Ge, and α-Sn.

Parameters	Si	Ge	α-Sn
a(nm)	0.54307^53^	0.56579^53^	0.6489^53^
C_11_(GPa)	167^48^	132^48^	69^48^
C_12_(GPa)	65^48^	49.4^48^	29^48^
m_c,Γ_(m_0_)	0.528^54^	0.038^54^	−0.058^54^
m_t,L_(m_0_)	0.133^57^	0.0807^58^	0.075^59^
m_l,L_(m_0_)	1.659^57^	1.57^58^	1.478^59^
γ_1_	4.22^60^	13.35^60^	−14.97^60^
γ_2_	0.39^60^	4.25^60^	−10.61^60^
γ_3_	1.44^60^	5.69^60^	−8.52^60^
E_p_(eV)	21.6^60^	26.3^60^	23.8^60^
a_c_(eV)	−10.06^53^	−7.83^53^	−6^53^
a_L_(eV)	−0.66^61,68^	−1.54^68^	−9.91^59^
a_v_(eV)	2.46^62^	1.24^62^	1.62^62^
b_v_(eV)	−2.1^62^	−2.9^62^	−2.01^62^
Δ_0_(eV)	0.044^63^	0.29^62^	0.8^62^
E_0_(eV)	4.093^11^	0.805^64^	−0.413^56^
E_L_(eV)	1.65^65^	0.664^66^	0.092^67^
E_v,max,_Γ(eV)	−0.55^69^	0^16^	0.53^16^
${\epsilon }_{{\rm{r}}}$	11.9^58^	16.2^58^	24^58^

## Materials Selection

**Figure 3:**
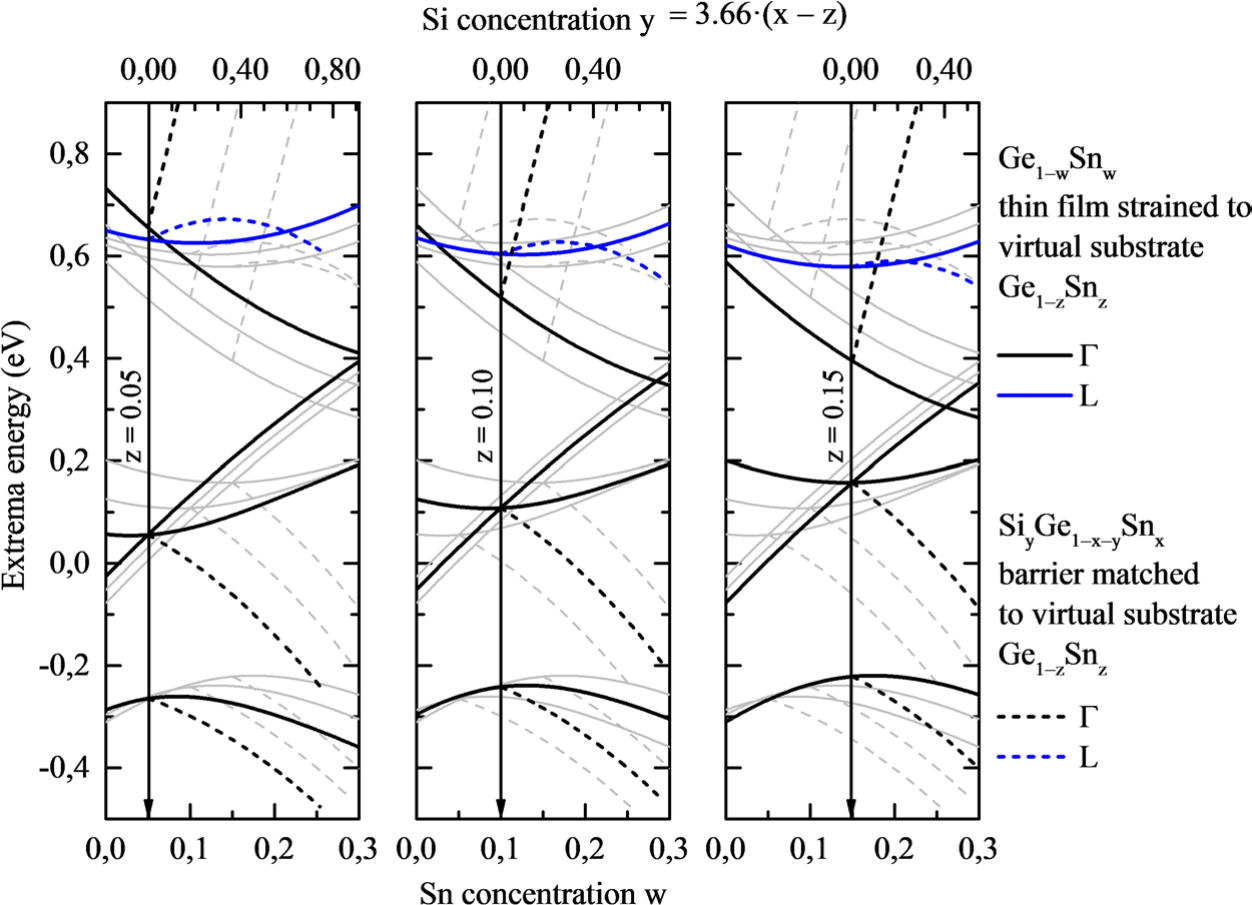
Conduction (Γ and L point) and the valence (HH, LH, and SO) band maxima for Ga_1−w_Sn_w_ QW material strained on virtual Ga_1-z_Sn_z_ substrate with z = 0.05, 0.10, and 0.15. Dashed lines show the conduction and valence band position for Si_y_Ge_1−x−y_Sn_x_ barrier lattice matched to the virtual substrate. Si concentration (y) in Si_y_Ge_1−x−y_Sn_x_ barrier is changing according to following formula: y = 3.66(x–z), which fulfills the condition of lattice matching to virtual Ga_1-z_Sn_z_ substrate. Grey lines on each panel are connected with blue and black lines on the other panels to make a comparison between all three cases easier.

The diagram shown in Fig. 2 is a starting point for the selection of materials for building a type I QW. From this diagram it is clearly visible that Ge_1-x_Sn_x_ with around 0.1 < x < 0.3 is a direct gap material which is a good candidate for QWs dedicated for light emitters. Direct gap materials are not necessary for barriers and SiGeSn is the most optimal choice in this case due to very flexible strain engineering in this alloy. Since the conduction and valence band positions move due to the built-in strain, different diagrams are necessary to evaluate the selection of materials for QW and barriers. Such diagrams are shown in Fig. 3. In this case the conduction (Γ and L point) band minima and the valence (HH, LH, and SO) band maxima are shown by solid lines for Ga_1−w_Sn_w_ QW material strained to virtual Ga_1-z_Sn_z_ substrate with z = 0.05, 0.10, and 0.15. In addition, the conduction and valence band positions for Si_y_Ge_1−x−y_Sn_x_ barrier lattice matched to the virtual substrate are shown in this diagram, see dashed lines. Si concentration (y) in Si_y_Ge_1−x−y_Sn_x_ barrier is changing according to following formula: y = 3.66(x-z), which fulfills the condition of lattice matching to virtual Ga_1-z_Sn_z_ substrate. When analyzing solid lines in Fig. 3 for the same bands (CB, HH, LH, or SO) it is clearly visible that the built-in strain strongly modifies the position of a given band and thereby can be used to engineer the bandgap in this QW system (for a better comparison all curves are plotted on each panel and curves corresponding to a given panel are thicker while the remaining curves are plotted by thin grey lines). In this case the strain-related shifts for CB, HH, LH, and SO band are calculated using the Bir-Pikus theory^72^, see proper formulas in the Methods section. So far it has been shown that this theory works very well in this material system in the range of low built-in strains^15,73^, i.e., the range which is considered in this work.

With the diagram shown in Fig. 3 it is relatively easy to identify compositions promising for type I Ge_1−w_Sn_w_/Si_y_Ge_1−x−y_Sn_x_/Ge_1−w_Sn_w_ QWs through identifying proper quantum confinement trends of the conduction and the valence band with the QW and/or the barrier content change. Thus, in the next part of the paper, the electronic band structure and gain spectra are discussed for sets of QWs built on the conclusions drawn from the diagrams shown in Fig. 3.

## Unstrained System

**Figure 4:**
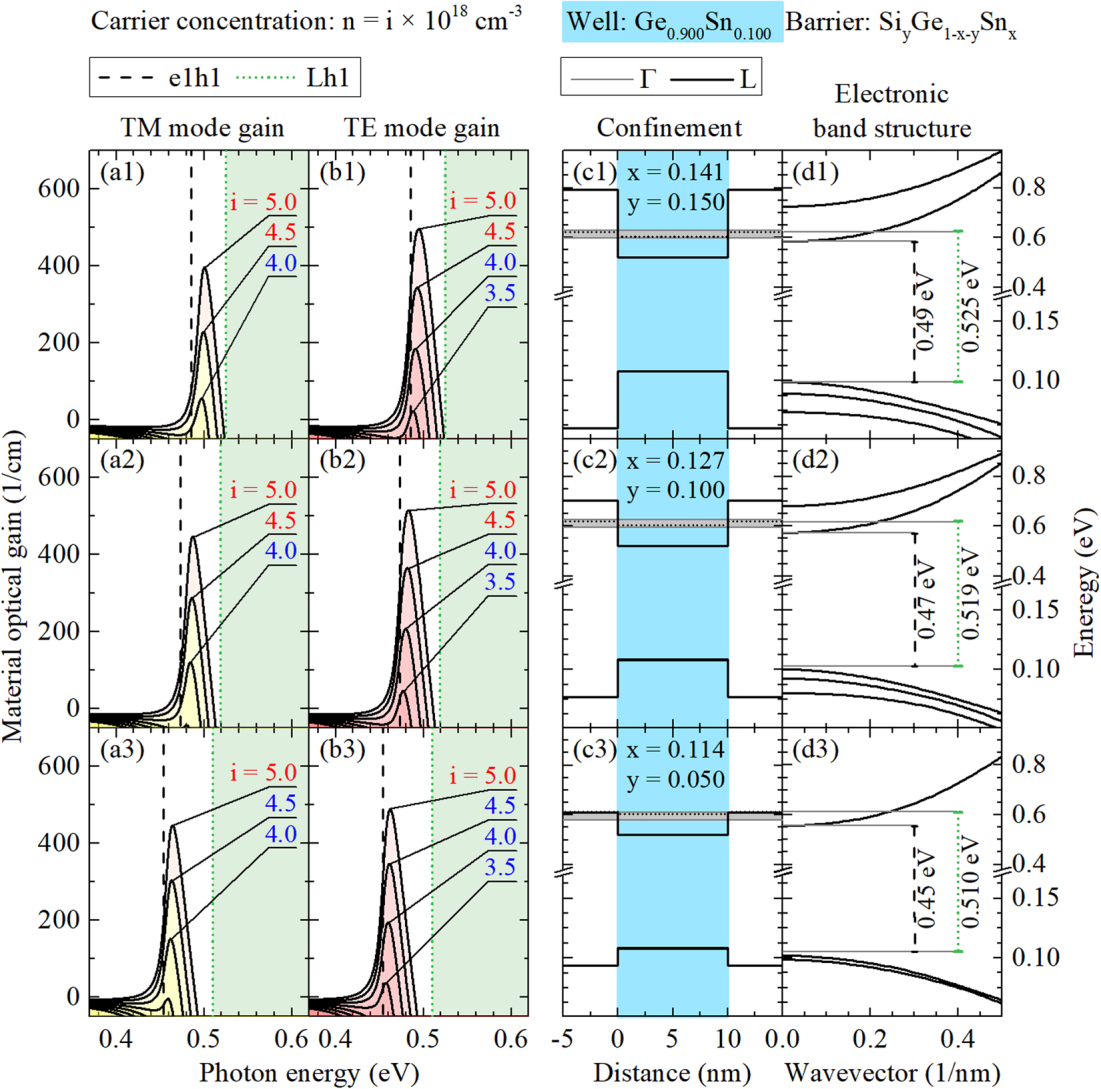
TE and TM modes of material gain spectra (left panels) and the electronic band structure (the quantum confinement potential and dispersions of electron and hole subbands, see right panels) for unstrained Ge_0.90_Sn_0.10_/Si_y_Ge_1−x−y_Sn_x_ QWs. In this case three Si_y_Ge_1−x−y_Sn_x_ barriers of various Si concentrations (y = 0.05, 0.10, and 0.15) are analyzed. Grey regions in confinement panels depicts QFLE value ranges for i from 2.0 to 5.0.

**Figure 5:**
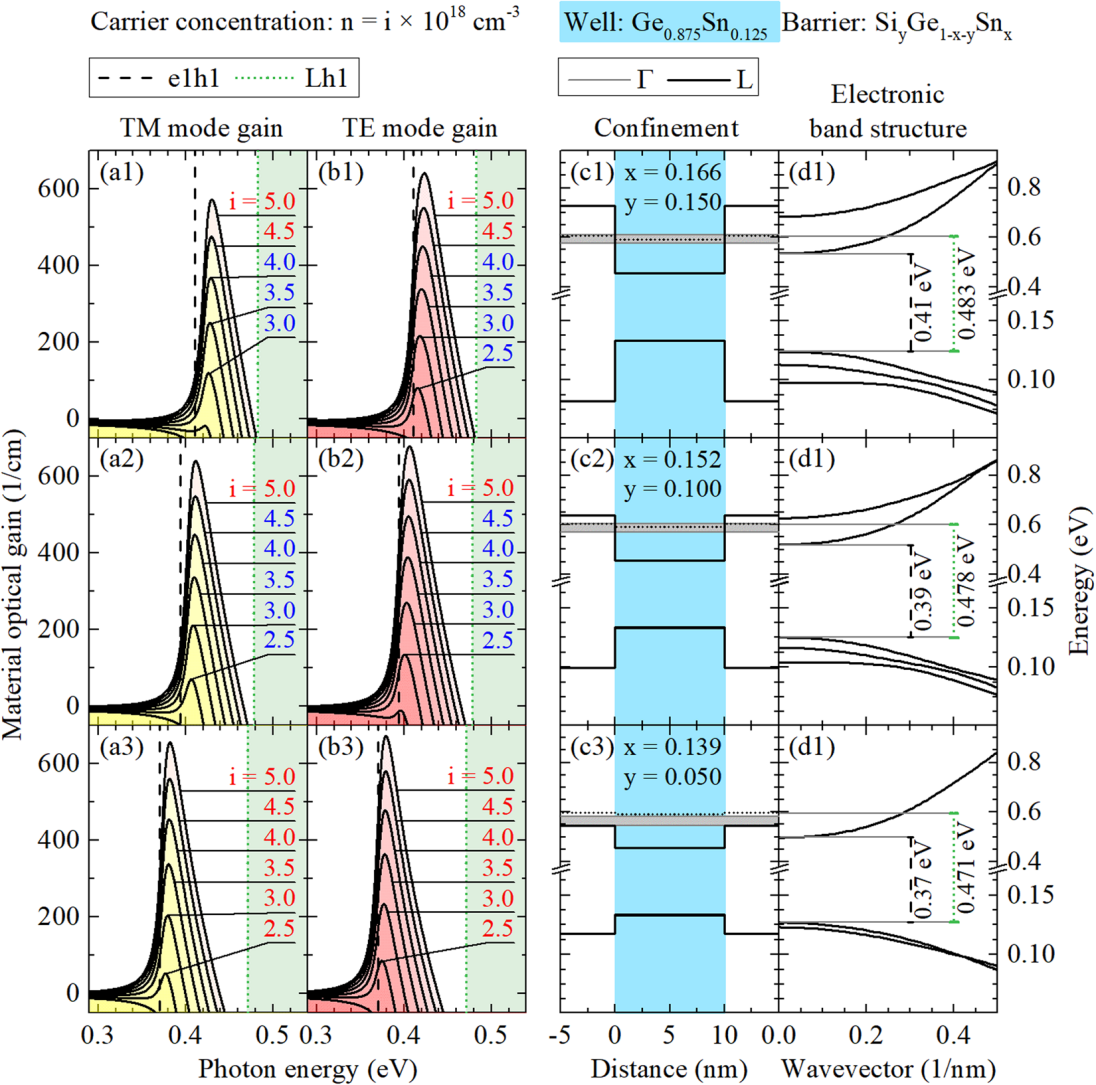
TE and TM modes of material gain spectra (left panels) and the electronic band structure (the quantum confinement potential and dispersions of electron and hole subbands, see right panels) for unstrained Ge_0.875_Sn_0.125_/Si_y_Ge_1−x−y_Sn_x_ QWs. In this case three Si_y_Ge_1−x−y_Sn_x_ barriers of various Si concentrations (y = 0.05, 0.10, and 0.15) are analyzed. Grey regions in confinement panels depicts QFLE value ranges for i from 2.0 to 5.0.

**Figure 6:**
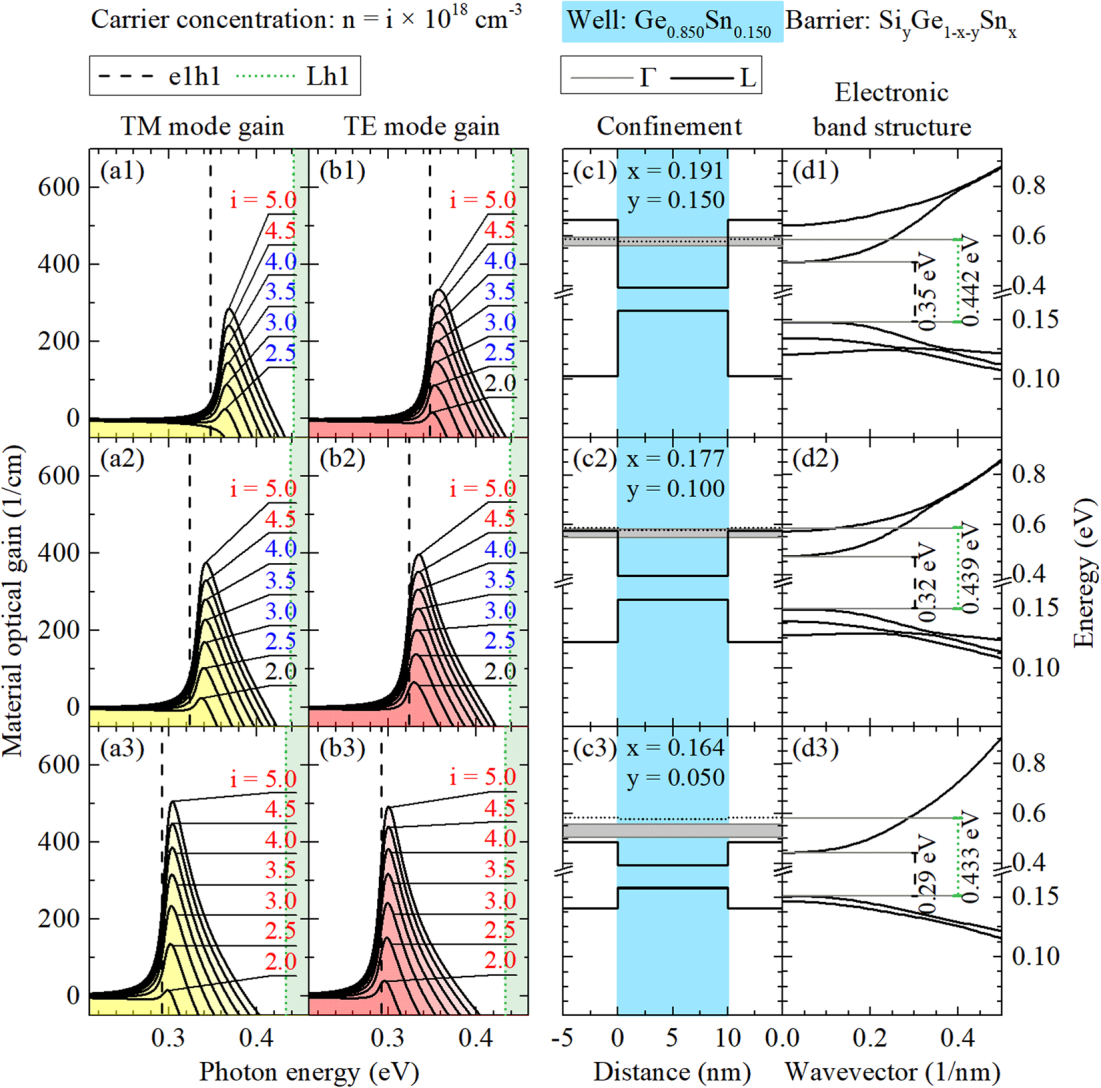
TE and TM modes of material gain spectra (left panels) and the electronic band structure (the quantum confinement potential and dispersions of electron and hole subbands, see right panels) for unstrained Ge_0.85_Sn_0.15_/Si_y_Ge_1−x−y_Sn_x_ QWs. In this case three Si_y_Ge_1−x−y_Sn_x_ barriers of various Si concentrations (y = 0.05, 0.10, and 0.15) are analyzed. Grey regions in confinement panels depicts QFLE value ranges for i from 2.0 to 5.0.

Figures 4, 5 and 6 show gain spectra (TE and TM mode) together with appropriate quantum confinements and electronic band structures of confined states for unstrained Ge_0.90_Sn_0.10_, Ge_0.875_Sn_0.125_, and Ge_0.85_Sn_0.15_ QW, respectively. In every case, three Si_y_Ge_1−x−y_Sn_x_ barriers of various Si concentrations (y = 0.05, 0.10, and 0.15) are analyzed. It is clearly visible that the increase in Si concentration enhances quantum confinement in the valence and conduction bands which is very favorable for laser applications (in general, a larger T_0_ temperature is expected for deeper QWs). However, this increase is connected with the increase in Sn concentration because of the lattice matching condition for this alloy. It is possible that the optical quality of lattice matched Si_y_Ge_1−x−y_Sn_x_ deteriorates with the increase in Si concentration and therefore Si_y_Ge_1−x−y_Sn_x_ barriers with large Si concentration are promising from the band structure point of view but can be difficult to manufacture from the technological point of view. Moreover it is worth noting that an electron leakage to the L point of the Brillouin zone in Si_y_Ge_1−x−y_Sn_x_ barriers can determine T_0_ in these QWs. The energy separation between the electron ground state and the L point is not very favorable in this case even for Si_y_Ge_1−x−y_Sn_x_ barriers with large Si concentration since the energy position of L point does not change significantly in this case, see Fig. 4. A significant electron confinement improvement is observed with the Sn concentration increase in the well layer, see the electronic band structure in Figs 4, 5 and 6. Thereby, an energy separation between the ground electron state energy in Ge_1−w_Sn_w_ QW and the L point energy in Si_y_Ge_1−x−y_Sn_x_ barrier significantly improves (and thereby a higher T_0_ is expected) for lasers containing Ge_1−w_Sn_w_ QWs with larger Sn concentration, but large T_0_ will be a challenge for this material system.

With the increase in Sn concentration in Ge_1−w_Sn_w_ QW, the gain spectra shift to red. For lower Sn concentration their intensities increase, which is connected with improving the confinement mentioned before, and decreases for higher Sn concentration, which seems to be connected with confined electrons effective mass decrease visible in conduction QW bands, see gain spectra in Figs 4, 5 and 6. It is also visible that, in case of the unstrained QWs, intensities of TE and TM modes are comparable and the every TM mode peak position is slightly blue-shifted in comparison to the corresponding TE mode peak position as it should be due to properties of heavy and light holes states. Moreover, the intensity of both modes of material gain decrease with the increase in Si concentration in Si_y_Ge_1−x−y_Sn_x_ barriers, which is hardly visible for lower and distinctly visible for cases with higher Sn concentration in QW.

Additional carrier current related information has been attached to Figs 4, 5 and 6. One may ask about values of quasi-Fermi levels energy (QFLE) of electrons and holes in presented unstrained QWs as an important parameter for designing real devices. In these QWs, all confined holes always seem to be close enough to valence band edge to not worry about them and leakage phenomena. Different situation appears in case of electrons which, for carrier concentrations chosen in calculations, seems to easily be able to fill almost all accessible confined states with energies below L valley energy. So some information, about how deep below L valley energy QFLE for electrons is set in every case, is marked by color of numbers which describes carrier concentrations, see Figs 4, 5 and 6. Red means that QFLE is higher than L valley energy. Blue means that QFLE is below L valley energy but not deeper than k_*b*_*T*, where T = 300 K. Black means that QFLE is more than k_*b*_*T* below L valley energy, where T = 300 K. To show QFLE value ranges in calculations for carrier concentrations from 2 · 10^18^ *cm*^−3^ to 5 · 10^18^ *cm*^−3^ there are grey regions put in charts with proper confinements.

Summarizing the results for unstrained Ge_1−w_Sn_w_/Si_y_Ge_1−x−y_Sn_x_/Ge_1−w_Sn_w_ QWs we conclude that the change of the content of virtual Ge_1-z_Sn_z_ substrate and the growth of unstrained Ge_1−w_Sn_w_/Si_y_Ge_1−x−y_Sn_x_/Ge_1−w_Sn_w_ QWs on such substrate (w = z) is a good strategy for tuning the emission wavelength of radiation with two comparable polarization components (TE and TM mode) possible to be emitted from this material system. In the next step, strain engineering in the QW region can be applied for tuning the contribution of TE and TM modes to lasing from the QW.

## Strain Engineering

**Figure 7:**
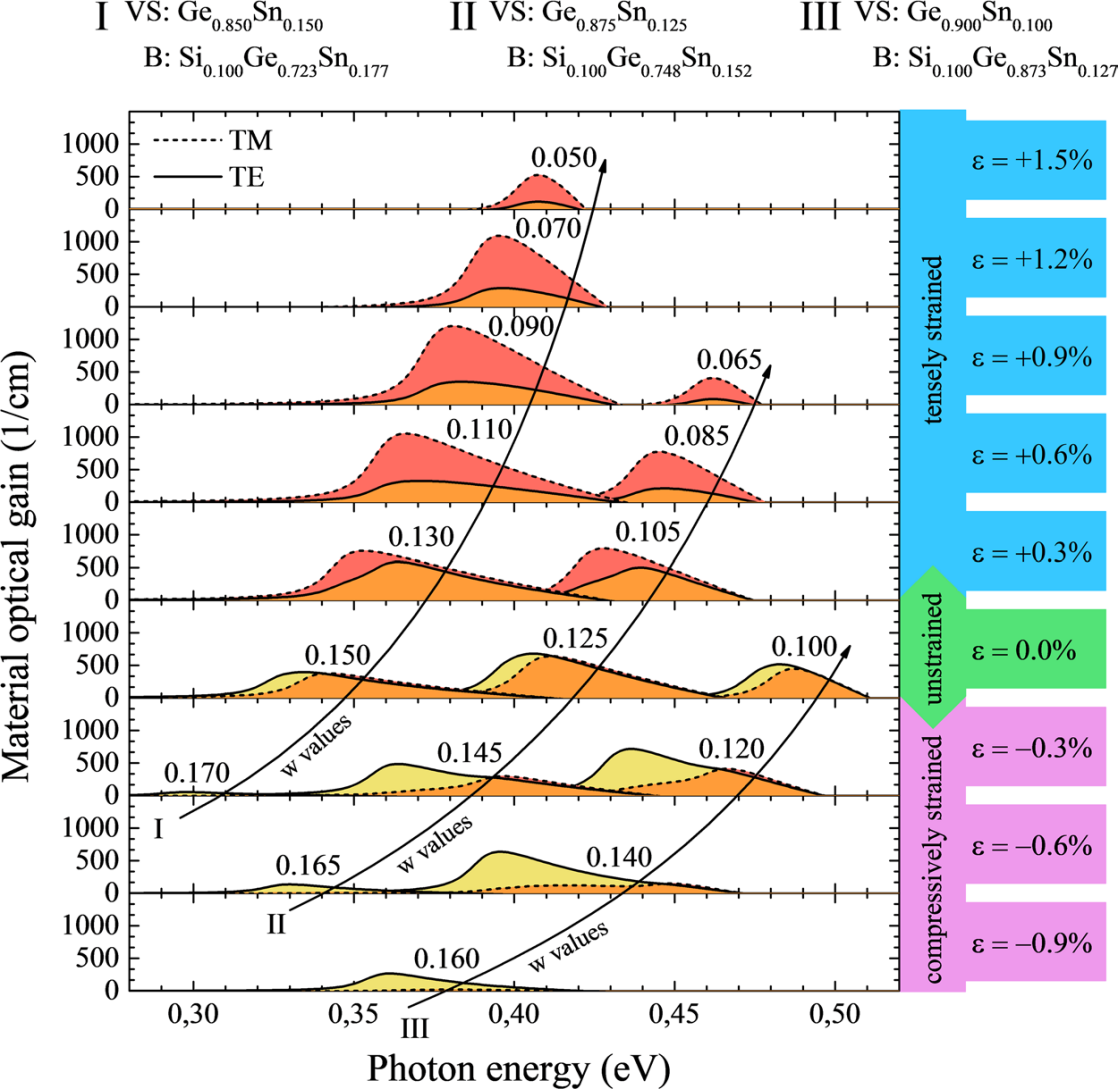
Spectra of TE (solid lines) and TM (dashed lines) mode of material gain for Ge_1−w_Sn_w_/Si_0.1_Ge_0.9-x_Sn_x_ QWs of various Sn concentrations (w) grown on virtual Ge_0.90_Sn_0.10_ Ge_0.875_Sn_0.125_, and Ge_0.85_Sn_0.15_ substrate.

Since, in case of unstrained QWs, highest gain values are obtained for QWs with barriers containing 10% of Si, those structures are expanded to the strained case by changing Sn concentration in QW layer. Figure 7 shows spectra of TE (solid lines) and TM (dashed lines) modes of material gain for Ge_1−w_Sn_w_/Si_0.1_Ge_0.9-x_Sn_x_/Ge_1−w_Sn_w_ QWs of various Sn concentrations (w) grown on virtual Ge_0.90_Sn_0.10_, Ge_0.875_Sn_0.125_, and Ge_0.85_Sn_0.15_ substrates. By changing the Sn concentration in Ge_1−w_Sn_w_ QW the built-in strain can be tuned from tensile to compressive. For tensile strained QWs the gain spectrum is dominated by the TM mode while for compressively strained QWs the TM mode of material gain is very weak and significantly blue-shifted in comparison to the TE mode. Therefore, for compressively strained Ge_1−w_Sn_w_/Si_0.1_Ge_0.9-x_Sn_x_/Ge_1−w_Sn_w_ QWs, emission of TE polarized light is expected, while a mixed light polarization (TE mode mixed with TM mode) is expected for tensile strained QWs. In addition, it is worth noting, that high dominating TE material optical gain is easier to achieve with virtual substrates containing less Sn (set along line III in Fig. 7) than in case when one may want to have dominating TM material optical gain (set along line I in Fig. 7).

**Figure 8:**
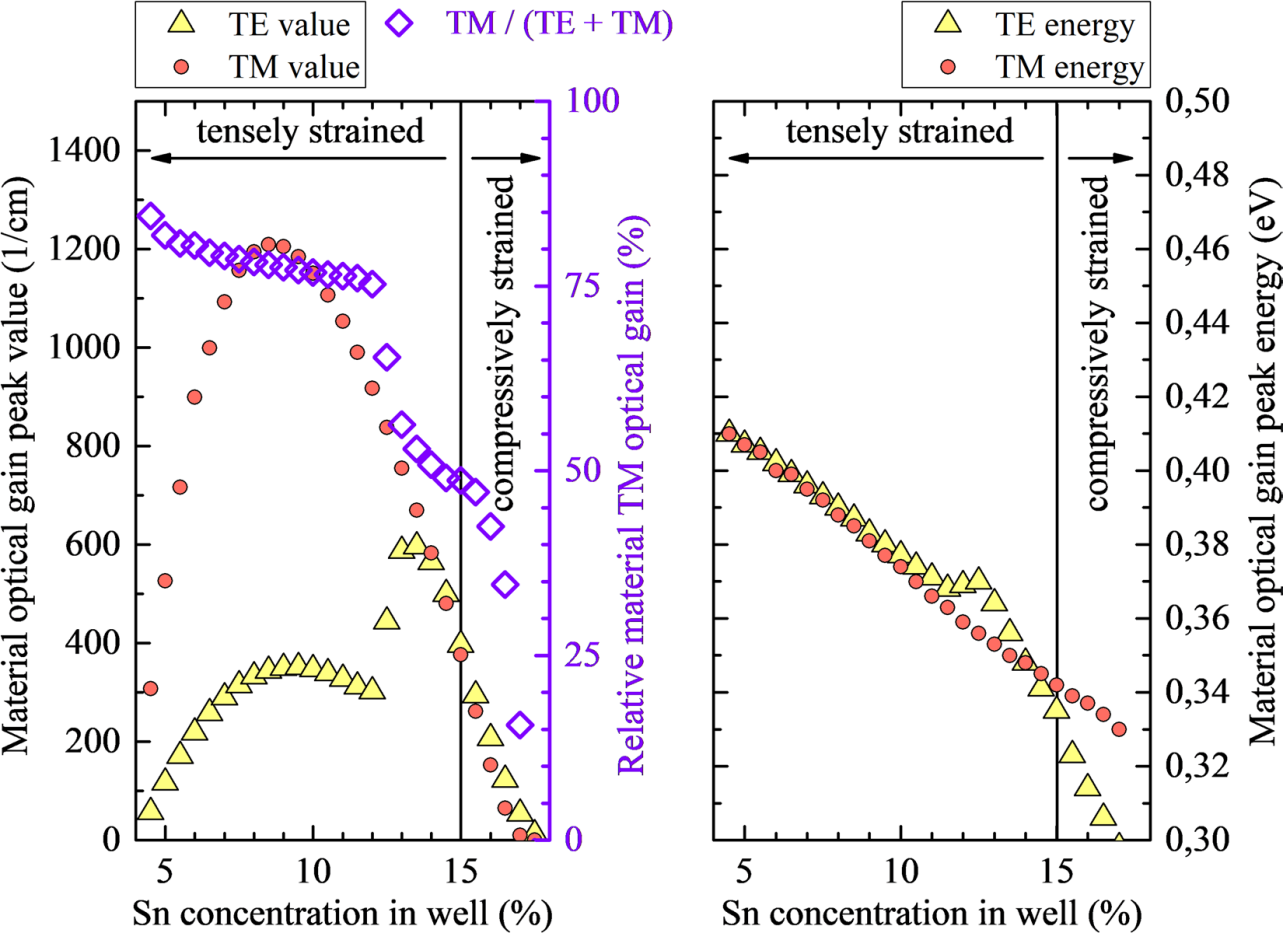
The peak position for TE and TM mode of material gain (right panel) the values of TE and TM mode at the peak TM energy together with the polarization degree (left panel) for Ge_1−w_Sn_w_/Si_0.1_Ge_0.723_Sn_0.177_ QWs grown on virtual Ge_0.85_Sn_0.15_ substrate.

Because there is less energetic separation between TE and TM modes of material gain spectra when the system is tensely strained, an analysis of optical gain peak values and positions for Ge_1−w_Sn_w_/Si_0.1_Ge_0.723_Sn_0.177_/Ge_1−w_Sn_w_ QWs grown on virtual Ge_0.85_Sn_0.15_ substrate (spectra along line I in Fig. 7) is presented below. In Fig. 8 TE and TM mode material gain peak positions are plotted in the left panel, values together with a TM polarization degree, both at TM gain peak energy are plotted in the right panel, where the polarization degree is defined as the ratio of the values of TM mode to the sum of the intensities of TE and TM mode at the given energy ((*polarization*(*E*) = *g*_*TM*_(*E*)/[*g*_*TE*_(*E*) + *g*_*TM*_(*E*)]). It is visible that TE peak changes its energy faster than TM peak with the less than 3% Sn concentration in the Ge_1−w_Sn_w_ QW changing (incorporating strain to QW layer). Situation is different for higher tensile strain, where both peaks go together and even have similar energy. This change in the TE mode material gain behavior is connected with less confined hole states number existing in the QWs with lower Sn concentrations and therefore also shallower confinements and higher tensile strain induced effects. In the right plot (Fig. 8) it is presented that calculated TM polarization degree increases from 50% to 80% with the tensile strain increase in the Ge_1−w_Sn_w_ QWs. In this case un-polarized light (50% polarization degree) is observed for unstrained QW and TM polarized light is observed for tensile strained QWs (75–80% polarization degree is observed for strains larger than 0.6%). The increase in polarization degree is mostly associated with the stronger TM mode and drop of TE mode material gain values for tensile strained QWs. The relative gain peaks spectral shift appearing due to the built-in strain is important in cases closer to unstrained situation only. In general, the last factor (the shift of TM and TE gain peaks) seems not to be controllable too much by the QW width when higher tensile strain situation is achieved. The reason is there is only one degenerated confined electron band and one degenerated confined hole band for these structures. However the less strain is present in the QW layer the more control of this factor should be apparent, since reducing strain in this system is connected with deepening its confinements in gamma point which increases number of bounded states. In order to study this phenomenon for the tensely strained QWs, gain spectra have been calculated with concerning various widths.

**Figure 9:**
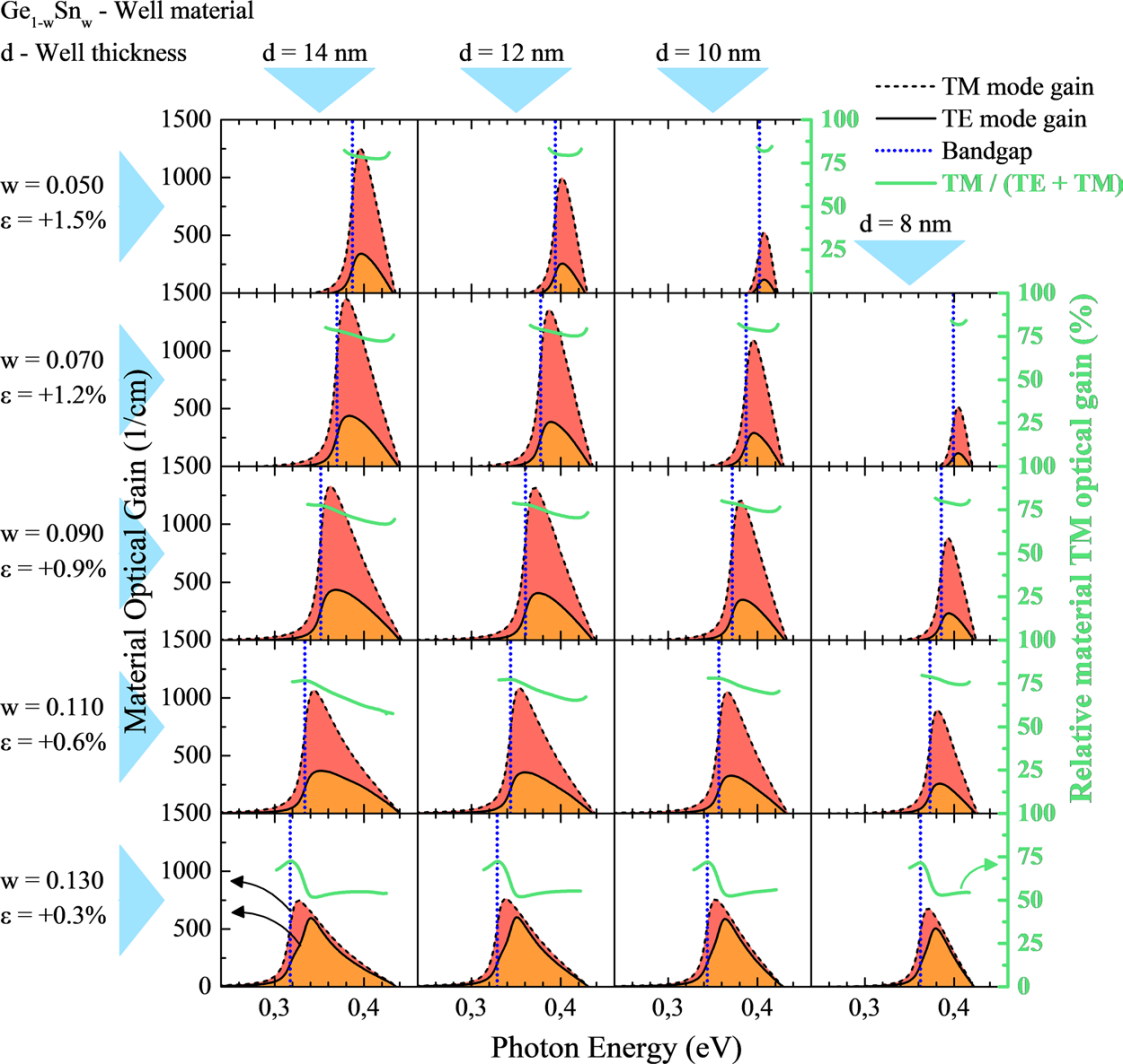
Spectra of TE (solid lines) and TM (dashed lines) mode of material gain for Ge_1−w_Sn_w_/Si_0.1_Ge_0.9-x_Sn_x_ QWs of various Sn concentrations and various widths grown on virtual Ge_0.85_Sn_0.15_ substrate.

Figure 9 shows spectra of TE (solid lines) and TM (dashed lines) mode of material gain for Ge_1−w_Sn_w_/Si_0.1_Ge_0.9-x_Sn_x_/Ge_1−w_Sn_w_ QWs of various widths grown on virtual Ge_0.85_Sn_0.15_ substrate. For all QW contents it is clearly visible that gain peaks shifts to blue by ~60 meV with the reduction of QW width from 14 to 8 nm, but differences between shifts of TE and TM peaks are negligibly small except case with low strain. With the QWs width, while the intensity of TE and TM modes almost does not change for QWs with larger Sn concentration, it decreases quite significantly for QWs with lower Sn concentration. It is visible, that calculated ratio between the values of TE and TM mode does not change significantly. Therefore the polarization degree should not vary with the QW width even if modes are changing their relative positions, see green lines for a given Sn concentration in Fig. 9. It means that the QW width does not significantly influence the mixing of TE and TM modes in this QW system, and the built-in strain together with number of confined states are the main factors which control the polarization degree of light emitted from Ge_1−w_Sn_w_/Si_y_Ge_1−x−y_Sn_x_/Ge_1−w_Sn_w_ QWs grown on virtual Ge_1-z_Sn_z_ substrates integrated with Si platform.

## Scattering of Material Parameters

Since some scattering of material parameters (i.e., bandgap bowing parameters) is present in the literature for (Si)GeSn alloys, see the previous discussion, it is interesting to analyze and discuss the influence of the accuracy of these parameters on our conclusions on light polarization engineering in this QW system. Such analysis has been performed changing the bowing parameter for the direct and indirect bandgap in GeSn by 20% (from 3.02 to 2.43 eV for the direct gap and from 1.23 eV to 0.98 eV for the indirect gap) and tuning the valence band offset by 20% (from 0.53 eV to 0.64 eV). It has been observed that these changes shift to blue the TE and TM peak positions but do not change their relative positions and intensities. Therefore the polarization degree of light does not change in this case. It allows us to claim that our conclusions on polarization engineering are valid for this material system even if the spectral position of the gain peak is predicted less accurately. It has been observed that the blueshift depends on QW width and content but in general this shift is smaller than 70 meV. Moreover it has been concluded that these changes do not influence trends in variation of the gain peak intensity with changes in Sn concentration in QW but slightly influence the range of QW contents for which a positive material gain is observed. Regarding changes in bowing parameters for SiSn, which were used for calculations of the bandgap for Si_y_Ge_1−x−y_Sn_x_ barriers, it has been observed that their influence on the spectral position and the intensity of TE and TM mode of material gain is negligible for deep QWs and starts to be important for shallow QWs. It suggests that results for Ge_1−w_Sn_w_/Si_y_Ge_1−x−y_Sn_x_/Ge_1−w_Sn_w_ QWs with Si rich barriers are more reliable.

Since the alloy disorder strongly influences the electronic band structure of highly mismatched alloys (HMAs), see for example ref.^74^, and GeSn is on the border of regular alloys and HMAs, it is interesting to know and comment how the alloy disorder is important for QWs containing GeSn. So far the influence of alloy disorder on the electronic band structure of GeSn was studied in ref.^16^. It was observed that there is no variation in the lattice constant but there is a significant difference in energy gap values when passing from the clustered to the uniform distribution. It is worth commenting that it can be one of the reasons for scattering the bowing parameter reported in the literature for the GeSn band gap. As mentioned before, bowing parameters used to our calculations are taken from ref.^16^ and correspond to uniform distribution of Sn atoms. In general such atom distribution is expected for the non-equilibrium growth process with the properly low growth temperature. An epitaxial growth at higher temperatures can lead to an atom segregation which is a well-known phenomenon even for regular semiconductor alloys like InGaAs^75^. In means that the regular atom distribution can be achieved in this alloy by lowering the growth temperature. On the other hand too low growth temperature leads to spontaneous formation of native point defects which are unfavorable for light emitters. According to motivation of this work the proper optimization of the growth temperature and/or other growth conditions/parameters is another challenge for this material system, but this issue is not out of the scope of this work.

## Conclusions

We have presented the first systematic study of light polarization engineering in Ge_1−w_Sn_w_/Si_y_Ge_1−x−y_Sn_x_/Ge_1−w_Sn_w_ QWs integrated with Ge_1-z_Sn_z_/Si platform. It has been shown how the emission wavelength in this system can be tuned by the content of virtual Ge_1-z_Sn_z_ substrate and the light polarization can be engineered via the built-in strain in Ge_1−w_Sn_w_ QW. Using virtual Ge_1-z_Sn_z_ substrate the built-in strain in Ge_1−w_Sn_w_ QW can be tuned from tensile (w < z) to compressive (w > z), changing the polarization degree from 80% TM to 100% TE polarization. Good quantum confinement for electrons and holes in this QW seems to be ensured by Si_y_Ge_1−x−y_Sn_x_ barriers lattice matched to Ge_1-z_Sn_z_ substrate. In addition, it is shown that the QW width should influence the degree of TM polarization for tensile strained QWs very weakly. We believe that the reported results will strongly motivate experimental studies of light polarization in GeSn-based QWs.

## Methods

### 8 band *k · p* Hamiltonian

For unstrained GeSn the 8 band ***k*** · ***p*** Hamiltonian is given below

2 $$H=(\begin{array}{llllllll}{E}_{CB}^{{\rm{\Gamma }}} & -\sqrt{3}{T}_{+} & \sqrt{2}U & -U & 0 & 0 & -{T}_{-} & -\sqrt{2}{T}_{-}\\  & {E}_{HH} & \sqrt{2}S & -S & 0 & 0 & -R & -\sqrt{2}R\\  &  & {E}_{LH} & Q & {T}_{+}^{\ast } & R & 0 & -\sqrt{3}S\\  &  &  & {E}_{SO} & \sqrt{2}{T}_{+}^{\ast } & \sqrt{2}R & -\sqrt{3}S & 0\\  &  &  &  & {E}_{CB}^{{\rm{\Gamma }}} & -\sqrt{3}{T}_{-} & \sqrt{2}U & -U\\  &  &  &  &  & {E}_{HH} & \sqrt{2}{S}^{\ast } & -{S}^{\ast }\\  &  &  &  &  &  & {E}_{LH} & Q\\  &  &  &  &  &  &  & {E}_{SO}\end{array})$$

The matrix elements in this Hamiltonian are the following:

3 $$\begin{array}{rcl}{E}_{CB}^{{\Gamma }} & = & {E}_{CB}^{{\Gamma }}(k=0)+\frac{{\hslash }^{2}}{2{m}_{0}}{s}_{C}({k}_{\parallel }^{2}+{k}_{z}^{2})\\ {E}_{HH}(k) & = & {E}_{HH}(k=0)-\frac{{\hslash }^{2}}{2{m}_{0}}(({\gamma }_{1}+{\gamma }_{2}){k}_{\parallel }^{2}+({\gamma }_{1}-2{\gamma }_{2}){k}_{z}^{2})\\ {E}_{LH}(k) & = & {E}_{LH}(k=0)-\frac{{\hslash }^{2}}{2{m}_{0}}(({\gamma }_{1}-{\gamma }_{2}){k}_{\parallel }^{2}+({\gamma }_{1}+2{\gamma }_{2}){k}_{z}^{2})\\ {E}_{SO}(k) & = & {E}_{SO}(k=0)+\frac{{\hslash }^{2}}{2{m}_{0}}{\gamma }_{1}({k}_{\parallel }^{2}+{k}_{z}^{2})\\ {T}_{\pm } & = & \frac{1}{\sqrt{6}}P({k}_{x}\pm i{k}_{y})\\ U & = & \frac{1}{\sqrt{3}}P{k}_{z}\\ S & = & \sqrt{\frac{3}{2}}\frac{{\hslash }^{2}}{{m}_{0}}{\gamma }_{3}{k}_{z}({k}_{x}-i{k}_{y})\\ R & = & \frac{\sqrt{3}}{2}\frac{{\hslash }^{2}}{2{m}_{0}}[({\gamma }_{2}+{\gamma }_{3}){({k}_{x}-i{k}_{y})}^{2}-({\gamma }_{3}-{\gamma }_{2}){({k}_{x}-i{k}_{y})}^{2}]\\ Q & = & \frac{-1}{\sqrt{2}}\frac{{\hslash }^{2}}{{m}_{0}}{\gamma }_{2}{k}_{\parallel }^{2}+\sqrt{2}\frac{{\hslash }^{2}}{{m}_{0}}{\gamma }_{2}{k}_{z}^{2}\end{array}$$

where the subscripts CB, HH, LH, and SO stand for the conduction (Γ point), heavy-hole, light-hole, and spin-orbit split off bands, correspondingly. *ℏ* is the Planck constant distributed by 2*π*, *m*_0_ is the electron mass, and ${k}_{\parallel }^{2}={k}_{x}^{2}+{k}_{y}^{2}$. *P* is the Kane matrix element defined as $P=-\,i\hslash /{m}_{0}\langle s^{\prime} |{p}_{v}|v\rangle$, where 〈*s*′| indicates a CB Bloch state of s-like symmetry and |*v*〉 is a VB *p* state with character |*v*〉 = |*x*〉, |*y*〉, or |*z*〉. Since the CB is preserved exactly in this Hamiltonian, the Luttinger parameters are modified, i.e. the parameters *γ*_1,2,3_ in the matrix elements are replaced by ${\gamma }_{1}\to {\gamma }_{1}-{E}_{P}/(3{E}_{g}^{{\rm{\Gamma }}})$ and ${\gamma }_{2,3}\to {\gamma }_{2,3}-{E}_{P}/(6{E}_{g}^{{\rm{\Gamma }}})$, where ${E}_{P}=2{m}_{0}P/{\hslash }^{2}$ is the Kane matrix element expressed in energy units. The term ${s}_{C}=\frac{1}{{m}_{c}^{\ast }}-({E}_{P}/3)[\frac{2}{{E}_{g}^{{\rm{\Gamma }}}}+1/({E}_{g}^{{\rm{\Gamma }}}+{{\rm{\Delta }}}_{SO})]$ replaces $1/{m}_{c}^{\ast }$ and is associated with the CB nonparabolicity. The energy scale is set in a way, where at the Γ point for unstrained Ge *E*_*HH*_(*k* = 0) = *E*_*LH*_(*k* = 0) = 0, *E*_*SO*_(*k* = 0) = Δ_*SO*_, and ${E}_{CB}^{{\Gamma }}(k=0)={E}_{g}^{{\Gamma }}$, where Δ_*SO*_ and ${E}_{g}^{\Gamma }$ are, respectively, the spin-orbit splitting and the energy gap for Ge.

### Built-in strains in (Si)GeSn

The strain-related shifts for CB and VB are considered using the Bir-Pikus Hamiltonian^72^. These strains change the quantum confinement potential for electrons and holes as shown in our earlier papers^42,43^. In the QW region the matrix elements of the 8-band ***k*** · ***p*** Hamiltonian are improved as below

4 $$\begin{array}{rcl}{E}_{CB}^{GeSn}(k) & \to  & {E}_{CB}^{GeSn}(k)+\delta {E}_{CB}^{hy}\\ {E}_{HH}^{GeSn}(k) & \to  & {E}_{HH}^{GeSn}(k)+\delta {E}_{VB}^{hy}-{\eta }_{ax}\\ {E}_{LH}^{GeSn}(k) & \to  & {E}_{LH}^{GeSn}(k)+\delta {E}_{VB}^{hy}+{\eta }_{ax}\\ {E}_{SO}^{GeSn}(k) & \to  & {E}_{SO}^{GeSn}(k)+\delta {E}_{VB}^{hy}+{\eta }_{ax}\\ Q & \to  & Q-\sqrt{2}{\eta }_{ax}\end{array}$$

where $\delta {E}_{CB}^{hy}=-\,2{a}_{C}(1-{c}_{12}/{c}_{11}){\varepsilon }_{xx}$, $\delta {E}_{VB}^{hy}=-\,2{a}_{V}(1-{c}_{12}/{c}_{11}){\varepsilon }_{xx},\,$and *η*_*ax*_ = −*b*_*ax*_(1 + 2*c*_12_/*c*_11_)*ε*_*xx*_ describe influence of the hydrostatic and shear strain components on the band structure. *a*_*C*_ and *a*_*V*_ are the hydrostatic deformation potentials for CB and VB, respectively; *c*_11_ and *c*_12_ are elastic constants, *b*_*ax*_ is the axial deformation potential and *ε*_*xx*_ is the in-plane strain in the GeSn layer. For GeSn QWs grown on virtual Ge_1-z_Sn_z_ substrate this strain is defined by the lattice parameters of Ge_1−w_Sn_w_ (QW material) and Ge_1-z_Sn_z_ substrate: ${\varepsilon }_{xx}=({a}_{G{e}_{1-z}S{n}_{z}}-{a}_{G{e}_{1-w}S{n}_{w}})/{a}_{G{e}_{1-w}S{n}_{w}}$. Material parameters for (Si)GeSn are determined consuming linear interpolation between parameters of relevant materials (Si, Ge, and Sn).

### Material gain

The optical gain for GeSn/SiGeSn QWs is designed for a given carrier density, which defines the quasi-Fermi levels ${E}_{c}^{F}$ and ${E}_{v}^{F}$ for the conduction and valence bands, correspondingly, and vice versa. The carrier density in a band in the QW is set by integration of result of the density of states, *ρ*(*k*), and the occupation probability of carriers (i.e. the Fermi-Dirac distribution) over the entire band. The Fermi-Dirac distribution for electrons (*f*_*c*_) and holes (*f*_*v*_) in the QW is given by Eqs (6) and (7)

5 $${f}_{c}({E}_{c}(k),{E}_{c}^{F})={(1+\exp (\frac{{E}_{c}(k)-{E}_{c}^{F}}{{k}_{B}T}))}^{-1}$$

6 $${f}_{v}({E}_{v}(k),{E}_{v}^{F})={(1+\exp (\frac{{E}_{v}(k)-{E}_{v}^{F}}{{k}_{B}T}))}^{-1},$$

where *k*_*B*_ is the Boltzmann’s constant and *T* is the temperature. The number of carrier in conduction (*N*) and valence (*P*) band is obtained by

7 $$N={S}_{well}[\sum _{{n}_{c}}{\int }_{0}^{{k}_{max}}{\rho }_{2D}(k){f}_{c}({E}_{{n}_{c}}(k),{E}_{c}^{F})dk]+{V}_{well}([{\int }_{0}^{{k}_{max}}{\rho }_{3D}(k){f}_{c}({E}_{CB}^{Ge}(k),{E}_{c}^{F})dk]\,+\,4[{\int }_{0}^{{k}_{max}}{\rho }_{3D}(k){f}_{c}({E}_{{{\rm{L}}}_{c}}^{Ge}(k),{E}_{c}^{F})dk])$$

8 $$P={S}_{well}[\sum _{{n}_{v}}{\int }_{0}^{{k}_{max}}{\rho }_{2D}(k){f}_{v}({E}_{{n}_{v}}(k),{E}_{v}^{F})dk]+{V}_{well}[\sum _{h}{\int }_{0}^{{k}_{max}}{\rho }_{3D}(k){f}_{v}({E}_{h}^{Ge}(k),{E}_{v}^{F})dk],$$

First relations on the right hand sides of equations (8) and (9) are related to the 2-D Fermi gases defined by electronic band structure of the QW obtained in the planewave expansion of the 8 ***k*** · ***p*** model, while the ones following are related to 3-D Fermi gases, which are defined by the electronic band structure of unstrained bulk Ge crystal designed using the 8 ***k*** · ***p*** model. *V*_*well*_ and *S*_*well*_ are correspondingly the QW volume and surface. The third part in integral (7) profits account of 3-D electron Fermi gas in L valley, where ${E}_{{{\rm{L}}}_{c}}^{Ge}(k)$ is parabolic approximation of this band in bulk crystal. It is shifted to the center of Brillouin zone in order to make simpler the quasi-Fermi level calculations and hence the factor 4 appears at this term (this explanation is equivalent to eight L valleys at the border of the Brillouin zone). Transitions related to this valley are omitted in the gain calculations. Further, for non-parabolic bands, integration is carried out in a ***k*** space with the density of states determined from the ***k*** · ***p*** calculations. *k*_*max*_ is the integration limit determined by convergence of the integrals (7) and (8).

A conventional method based on the relaxation time approximation convoluted with a Lorentzian function ($L({n}_{c},{n}_{v},k)=\frac{\hslash /{\tau }_{b}}{{\rm{\Delta }}{({n}_{c},{n}_{v},k)}^{2}+{(\hslash /{\tau }_{b})}^{2}}$, where *ℏ* is Plank’s constant and Δ is the proper energy difference) with a proper broadening time (*τ*_*b*_ = 0.1 ps) has been used to calculate the gain spectra^42,43^. In this approximation the transverse electric (TE) and transverse magnetic (TM) gain is given by Eq. (9)

9 $${g}_{\beta }^{TE(TM)}(\hslash \omega )={C}_{0}{\beta }^{-1}\sum _{{n}_{c},{n}_{v}}\int dk({f}_{{n}_{c}}(k)-{f}_{{n}_{v}}(k))\sum _{i}{M}_{ci}^{TE(TM)2}{|{I}_{{n}_{v}i}^{{n}_{c}}(k)|}^{2}L({n}_{c},{n}_{v},k)$$

where ${C}_{0}={q}^{2}/(\omega {m}_{0}^{2}\tau c{\varepsilon }_{0})$(*q* - elementary charge; *m*_0_- electron mass; *ω*– angular frequency, *τ*- time constant; *c*– speed of light; *ε*_0_- dielectric constant), *i* represents the heavy and light hole subbands, *β* in this case is the propagation constant of the TE and TM mode, ${M}_{ci}^{TE(TM)}$ is the matrix element of TE (TM) mode, and ${I}_{{n}_{v}i}^{{n}_{c}}(k)$ is the overlap integral.
